# Ultrasound location of pulmonary nodules in video‐assisted thoracoscopic surgery for precise sublobectomy

**DOI:** 10.1111/1759-7714.13384

**Published:** 2020-03-17

**Authors:** Yue‐Long Hou, Yan‐Dong Wang, Hong‐Qi Guo, YuKun Zhang, YongKuan Guo, HongLi Han

**Affiliations:** ^1^ Department of Thoracic Surgery Tianjin Third Central Hospital Tianjin China; ^2^ Tianjin Institute of Hepatobiliary Disease Tianjin China; ^3^ Tianjin Key Laboratory of Artificial Cell Tianjin China; ^4^ Artificial Cell Engineering Technology Research Center of Public Health Ministry Tianjin China; ^5^ Department of Ultrasound Tianjin Third Central Hospital Tianjin China

**Keywords:** Location, pulmonary nodule, sublobectomy, video‐assisted thoracoscopic ultrasound

## Abstract

**Background:**

We investigated the clinical value of accurate sublobectomy of pulmonary nodules using video‐assisted thoracoscopy (VATS). In June 2017 to June 2019, single lung nodule patients who accepted thoracoscopic resection were included. Palpation and intraoperative ultrasound (IU) were used to localize lung nodules, and the success rate, location time and safety compared. Performance of lung nodule ultrasound was assessed. The success rate of IU localization of pulmonary nodules with different properties was studied.

**Results:**

A total of 33 cases with single pulmonary nodules were included in the study, and 32 cases (97%) were successfully located by IU as opposed to 16 cases (48.5%) located by palpation (*P* < 0.05). Clear hypoechoic ultrasound images of nodules were obtained in all 32 cases, and the diameter of pulmonary nodules on ultrasound and CT were found to have a significant correlation (R = 0.860, *P* = 0.000). The average positioning time of IU was lower than that of the palpation group (*P* < http://0.05.no complications occurred during ultrasound examination. The success rate of intraoperative ultrasonic localization between the pure ground‐glass opacity (p‐GGO) group and the mixed‐ground‐glass opacity (m‐GGO) group was 90%, 100% (*P* = 0.526).

**Conclusions:**

In thoracoscopic surgery, IU can locate pulmonary nodules accurately, efficiently and safely, and also has a high degree of accuracy in locating different types of pulmonary nodules.

## Introduction

Lung cancer screening test results in the United States suggest that low‐dose computed tomography (CT) screening in high‐risk lung cancer population, not only increased the detection rate of early lung cancer, but also reduced the death rate by 20%.^1^ With the current wide use of chest CT, more pulmonary nodules are being confirmed by CT examination; however, some are considered malignant or there is the possibility of malignancy, and they need to be surgically removed.^2^ Nowadays, minimally invasive treatment technologies including thoracoscopy and da vinci robot are widely used in clinic. What is worth noting is that accurately locating pulmonary nodules is the key for the invasive procedure, for avoiding extended resection, or thoracotomy.^3^

Many pre‐ or intraoperative location techniques have been reported in previous studies such as CT‐guided hook‐wire localization, methylene blue or isotope injection, and intraoperative magnetic resonance (iMR) imaging navigation, etc. Although of high accuracy, problems such as pneumothorax, guide pin displacement and dye diffusion are evident.^4^, ^5^, ^6^ In this study, ultrasound location combined with thoracoscopic surgery in pulmonary nodule resection was utilized in order to observe the safety and feasibility of intraoperative ultrasound (IU).

## Method

### Patients

Patients who underwent thoracoscopic surgery between June 2017 and June 2019 in the Department of Thoracic Surgery of The Third Central hospital of Tianjin due to isolated pulmonary nodules were enrolled into the study. Entry criteria were as follows: (i) patients with suspected malignant pulmonary nodules less than 2 cm, confirmed by performing thin‐slice chest CT; (ii) no signs of previous malignant disease; (iii) all patients cardiopulmonary function was able to tolerate surgical removal of segmentectomy or lobectomy, and (iv) patients had not previously had a biopsy, localization and treatment before operation. Exclusion criteria: (i) patients with nodules close to the hilar zone where it is difficult to perform a wedge resection, and (ii) no obvious pleural traction signs found by thoracoscopy.

### Surgical procedure

**Figure 1:**
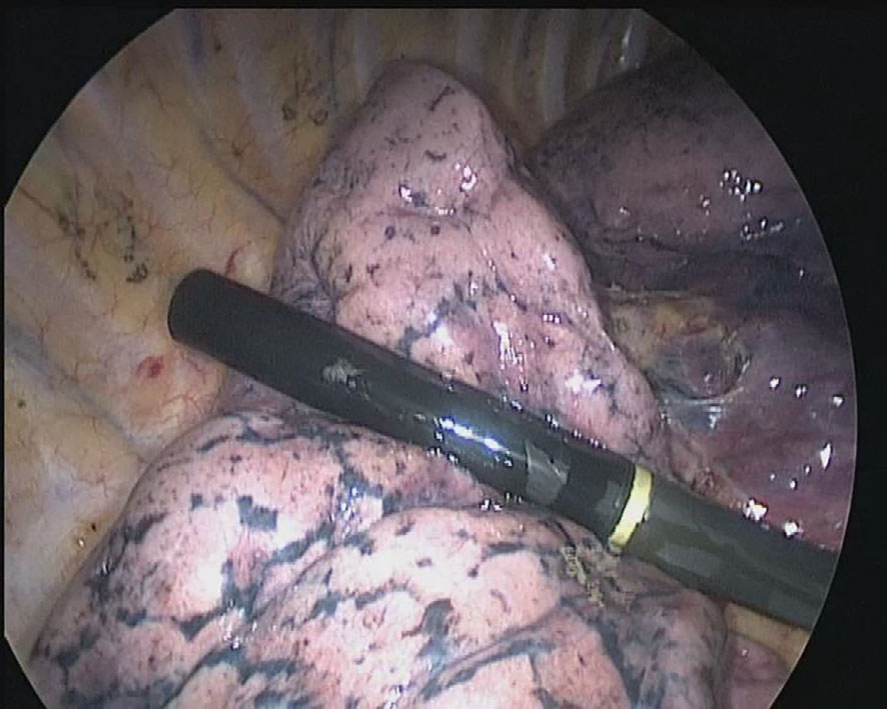
Intraoperative ultrasound localization of pulmonary nodules.

Patients were placed in the full lateral decubitus position, general anesthesia was induced, and double‐lumen endotracheal intubation with contralateral single lung ventilation was performed. Location of the cannula was confirmed by fiberoptic bronchoscopy with unilateral pulmonary ventilation. After insertion of the trocar in the seventh intercostal space in the midaxillary line and a 5 mm posterior axillary line trocar, CO_2_ insufflation was delivered at a low flow rate to obtain a constant pressure of 6 mmHg to promote lung collapse. The time and complications of lung collapse were recorded. A 3 cm access incision was made in the fourth intercostal space on the anterior axillary line and a retractor/protector was placed. Gauze was then clamped using a round oval clamp to gently press the lesions in the lung, and the lesions labeled by palpating the nodules. The palpation method was considered to have failed if it took more than 12 minutes. Pulmonary nodules were located using a L10‐4lap linear probe (10 mm in diameter, 35 cm in length, multifrequency 7.5–10 MHz array transducer, detection depth 13 cm, scanning angle 60 degrees, focal length 6–90 mm) with a Philips CX50 color Doppler diagnostic ultrasound by a senior ultrasound doctor. The probe was inserted into the chest through the operating hole, and the costal, mediastinal and diaphragmatic surfaces of the lung were explored (Fig 1). The pulmonary nodules were subsequently labeled and their characteristics under ultrasound‐guidance recorded. The surface of the nodules was cauterized using a cautery stick, and a wedge resection with a 2 cm margin was performed. The specimen was then sent to the department of pathology for frozen section histopathology to confirm the accuracy of excision. Surgical procedures consisted of lobectomy or pneumonectomy with mediastinal node dissection according to the pathology results.

**Figure 2:**
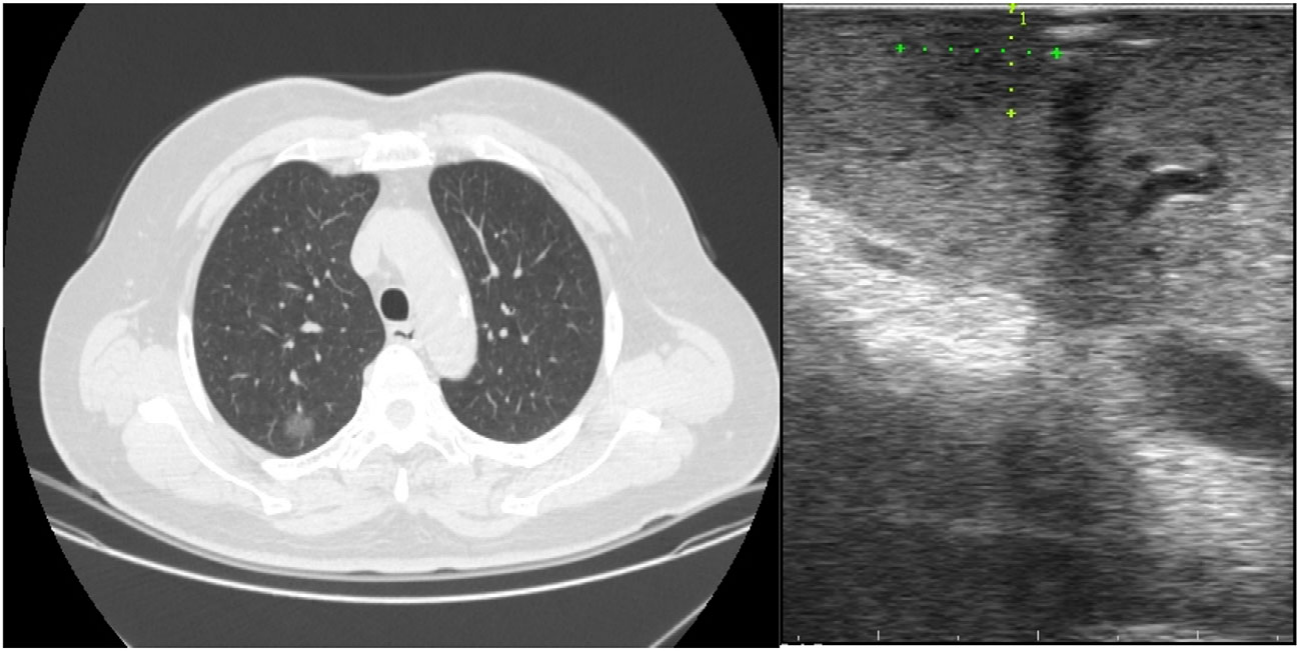
(**a**) CT scan and (**b**) ultrasound which confirmed the presence of pure ground‐glass nodules.

### Statistical analysis

All data are presented as mean ± standard deviation. Fisher's exact probability method was used to compare the success rate of the two methods and the subgroup. The time of the two groups was found to be normal distribution. Paired sample *t*‐test and a Pearson's correlation test were used for comparison. *P*‐values less than 0.05 indicated a statistically significant difference.

## Results

**Figure 3:**
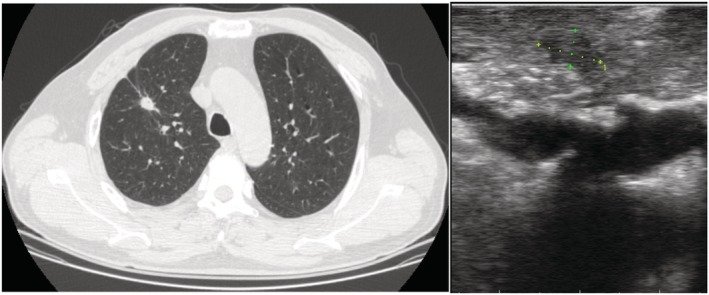
(**a**) CT scan and (**b**) ultrasound manifestations of pulmonary nodules.

**Figure 4:**
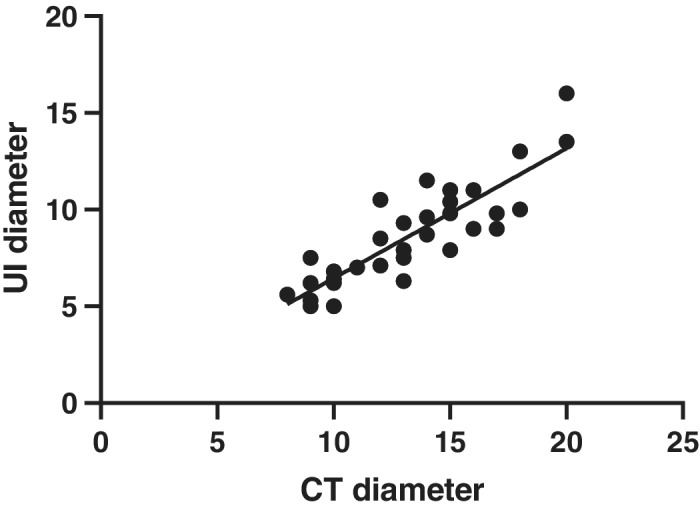
Correlation coefficient between pulmonary nodule diameter on CT scan and ultrasound, R = 0.860, *P* < 0.05.

A total of 33 patients were assessed for eligibility, including 19 men and 14 women, with an average age of 58.12 ± 9.49 (range 35–73 years). There were 10 nodules located in the right upper lobe, four in the middle lobe, four in the lower lobe, and six in the left upper lobe. The mean diameter of pulmonary nodules was 13.21 ± 3.37 mm (range 8–20 mm), mean distance to pleura was 14.64 ± 5.58 (range 5–28 mm) (Table 1). There were no complications (arrhythmia, or significant change of blood pressure) in all 33 patients when an artificial pneumothorax was applied. The mean time of artificial pneumothorax lung collapse was 3.42 ± 1.27 minutes (range 1.5–7 minutes). A total of 32 patients' lesion were located by ultrasound (97%), which was significantly higher than the 16 cases (48.5%) by palpation (*P* < 0.05, Table 2). A total of 32 cases had a clear hypoechoic ultrasound image (Figs 2 and 3) and the mean diameter was 8.69 ± 2.60 (range 13.5–5 mm), and CT detection of nodal diameter was 13.34 ± 3.33 mm (8–20 mm) which had a significant correlation with ultrasound image (r = 0.860, *P* = 0.000) (Fig 4).

**Table 1:** Characteristics of pulmonary nodules

Pulmonary nodules					
Size (diameter under CT)	Range		Mean diameter		
	8‐20 mm		13.21 ± 3.37 mm		
Distance from pleura (under CT)	Range		Mean depth		
	5‐28 mm		14.64±5.58 mm		
Modules characteristics	p‐GGO	m‐GGO		Solid pulmonary nodules	
	10	9		14	
Location	The upper lobe of right	The middle lobe of right	The lower lobe of right	The upper lobe of left	The lower lobe of left
	10	4	9	4	6

**Table 2:** Comparison of success rates

	Succeed	Fail	Success rate
Method			
Palpation	16	17	48.5%
Intraoperative ultrasound	32	1	97%

There was a statistical difference in the success rate between the two groups. *P* < 0.05.

**Figure 5:**
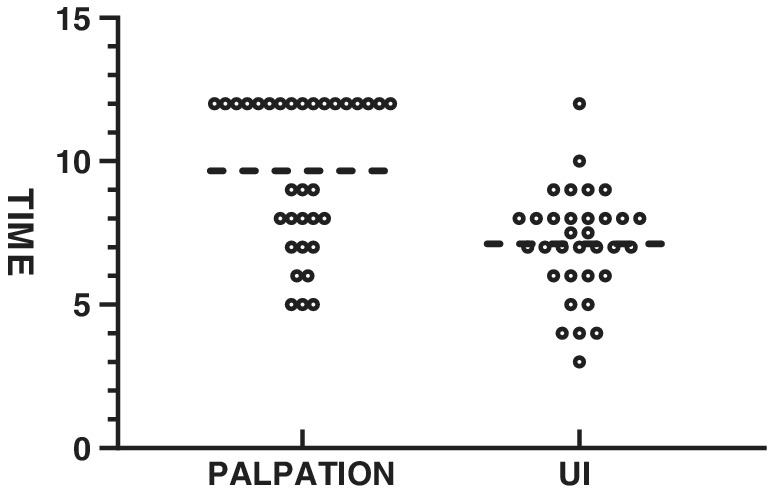
Pulmonary nodule localization time. Palpation represents the palpation group. UI represents the intraoperative ultrasound group. *P* < 0.05.

The average positioning time of IU was lower than that of the palpation group (7.12 ± 1.87 vs. 9.66 ± 2.62, *P* < 0.05) (Fig 5). No arrhythmia, bleeding and other complications occurred during the ultrasound examination. Pulmonary nodules were divided into the solid pulmonary nodules group (14), the pure ground‐glass opacity (p‐GGO) group (10 cases) and the mixed‐ground‐glass opacity (m‐GGO) group (nine), and the success rate of intraoperative ultrasonic localization was 90% and 100%, respectively between the p‐GGO group and the m‐GGO group (*P* = 0.526) (Table 3).

**Table 3:** Intraoperative ultrasound was used to locate the pulmonary nodules

	Succeed	Fail	Success rate
Nodule type			
p‐GGO	9	1	90%
m‐GGO	9	0	100%

p‐GGO, pure ground‐glass nodules; m‐GGO, mixed ground glass nodules group. There was no significant difference in success rate between the two groups. *P* = 0.526.

One case of emphysema, with a 10 mm nodule that was located 10 mm under the visceral pleura, could not be found either by ultrasound or palpation. After CT scan confirmation that the lesion was located in the lingual segment of the left upper lobe, tongue segment resection was performed and the pathological result was carcinoma in situ.

**Figure 6:**
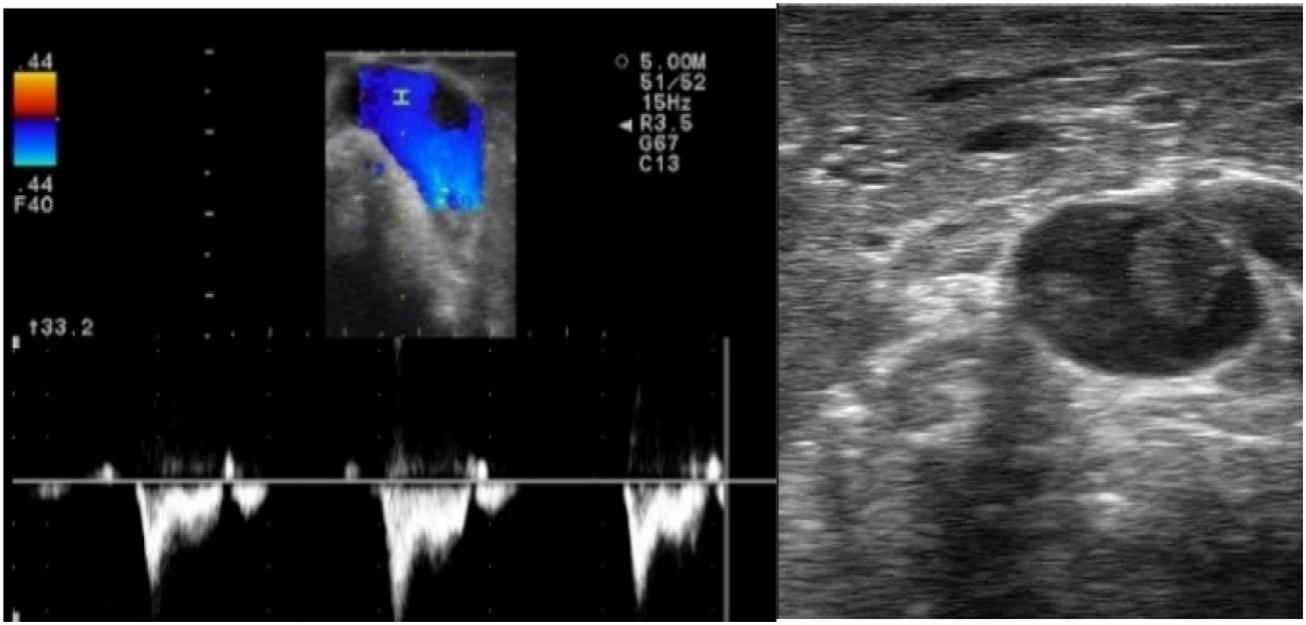
Intraoperative ultrasonography of pulmonary embolism. (**a**) Doppler image. The visible filling defect is a thrombus. (**b**) A clot can be seen in the blood vessel.

Pulmonary artery thrombosis was found in one patient during nodule localization examination, which was not found in preoperative enhanced CT text (Fig 6). Positive anticoagulant and antithrombotic treatment was given for 14 days after surgery and the thrombus disappeared after re‐examination on chest enhanced CT. The patient was eventually discharged.

Of 33 nodules examined, there were 14 that were malignant such as adenocarcinoma, squamous cell carcinoma, AIS etc, and 19 benign that included AAH, hamartoma, pulmonary fibrosis and fungal infection.

## Discussion

Ultrasound examination is widely used in abdominal organs with advantages such as noninvasive frontal, economical, real‐time, accuracy and avoidance of complications.^7^ However, such technology was rarely applied to lung examination due to the high attenuation in lung tissue before. With the development of technology, ultrasound has been found to be useful for positioning in abdominal surgery, and this method is now being used in chest surgery.^8^, ^9^ In the study by Wada *et al*. agar was injected into the lung of a pig to create a false tumor. The results showed ultrasound could clearly display the location of pulmonary pseudotumor and the small blood vessels and trachea in the lung tissue. Ex vivo, pulmonary nodules were examined by ultrasound, and the test results indicated that complete pulmonary collapse could be visualized by ultrasound.^11^ The above findings are the theoretical basis for this study.

Pulmonary collapse is an important factor in nodule localization. Ujiie *et al*.^11^ found some of the nodules were difficult to detect in the semiexpanded state of the lungs because of the presence of air around the tumor. Patients with asthma and COPD were excluded in the study of the application of ultrasound localization in pulmonary nodule surgery by Kondo *et al*. because they concluded that ultrasound was more difficult when residual air was present during the operation.^12^ In this study, low flow and low pressure (8 mmHg) was injected into the chest in order to make the lung collapse to assist with the ultrasonic examination. In addition, there was no hemodynamic‐related complications observed which is consistent with those of previous studies.^10^, ^13^

Matsumoto *et al*.^14^ successfully located 25 pulmonary nodules by IU, even in three cases a nodule was neither visible nor palpable. Khereba *et al*.^3^ located 43 of the 46 pulmonary nodules by IU with a success rate of 93%. In this study, 33 cases of intraoperative ultrasonic localization of pulmonary nodules were enrolled within two years. The mean size of nodules was 13.21 ± 3.37 mm (8–20 mm), and the distance from the pleura was 14.64 ± 5.58 mm. The results showed that 16 pulmonary nodules were successfully located in the palpation group (48.5%), and 32 in the IU group (97%). Based on the results, the IU group was significantly higher than that in the palpation group (*P* < 0.05). Although there is no direct comparison with ultrasound using preoperative positioning methods such as CT‐guided hook‐wire metal localization, injection of methylene blue or isotopes, and intraoperative tracheal endoscopic magnetic navigation, our success rate was no lower than these methods, there was no additional radiation and the method used in this study is simple and more economical.^4^, ^5^, ^6^

In this study, the palpation method was considered to have failed if it lasted for more than 12 minutes. The reasons for this were: (i) the previous clinical experience of our center suggested that the longer the palpation time, the lower the probability of finding pulmonary nodules, and (ii) previous studies have suggested that excessive surgical procedures increase the risk of tumor metastasis^15^, ^16^ and prolong the surgical time. According to the results, intraoperative ultrasonic localization time was significantly shorter than that of the palpation method (7.09 ± 1.80 minutes 9.67 ± 2.62 minutes: *P* < 0.05). The positioning time is consistent with Khereba *et al*. who indicated that IU can locate pulmonary nodules with high accuracy and within a short time which creates the conditions for minimally invasive surgery.

None of the 33 patients had any arrhythmia, bleeding, blood pressure changes or other complications during IU examination, suggesting that IU is a safe technique, which is consistent with many of the above findings. There are of course risks in this approach, and it is limited to patients without complete pulmonary collapse and an experienced ultrasonographer is always required.

Many solid pulmonary nodules can be easily visualized by IU due to the density difference from adjacent normal parenchyma, showing a hypoechoic pattern, with an associated acoustic enhancement of the posterior echo.^17^ However, the internal echo images under ultrasound are hyperechoic or hypoechoic, which are not consistent with their size, histologic type, or presence of solid components. The echo types are closely correlated to the degree of pulmonary collapse.^12^ In this study, the mean longest diameter of pulmonary nodules under ultrasound was significantly correlated with the mean diameter under CT (R = 0.860, *P* < 0.05).

Intraoperative ultrasound has been used for the localization of solid pulmonary nodules, which are easily visualized, even in a completely collapsed lung.^17^ In this study, all 14 cases of solid pulmonary nodules were successfully identified. However, pulmonary ground‐glass nodules, especially p‐GGO, can be difficult to identify using IU because p‐GGO lesions have a similar density to adjacent normal parenchyma and are easily disturbed by surrounding air making them difficult to identify. Experimental studies have evaluated the possibility of detecting small lung nodules including GGO. The researchers seeded different patterns of echogenicity target nodules to porcine lung tissue to simulate ground‐glass pulmonary nodules for ultrasonic examination and the sensitivity rate reached 86.6%.^18^ In addition, the researchers conducted ultrasonic examination of the GGOs resection in vitro, and all six p‐GGOs and 11 m‐GGOs were successfully identified.^19^ The above studies suggest that the GGO can be identified by ultrasound. In this study, there were 10 cases in the p‐GGO group and nine cases in the p‐GGO group. The localization success rate of the two types of pulmonary nodules by IU was 90% and 100% respectively, suggesting that IU could accurately locate both types of nodules as long as it is within a certain range of size and depth.

In one case of emphysema, the p‐GGO nodules with a diameter of 8 and 27 mm under the visceral pleura could not easily be detected by ultrasound and palpation. CT confirmed that the lesion was located in the left upper lobar lingual segment, and tongue segment resection was performed. The pathological result was carcinoma in situ.

By using ultrasound, Piolanti *et al*.^20^ found nodules that had not been visible on CT scan, while there were no such findings in this study. During intraoperative inspection, a 58‐year‐old male patient, previously diagnosed with pulmonary nodules, was found to have a pulmonary embolism. This patient had no previous evidence of cardiovascular disease, as preoperative enhancement CT scan had shown no evidence of a pulmonary thromboembolism. Following the postoperative pathological results in the pneumonia lesions, and after normal antithrombotic therapy, the patient was eventually discharged safely, thus avoiding the risk of surgery.

The deficiency in our study is the limited number of cases included which affects the results statistically, and therefore more studies may be required in the future to confirm the results.

In conclusion, low pressure and low flow of CO_2_ injection into the thorax is a safe way to induce lung collapse. Thoracoscopic ultrasound can locate pulmonary nodules safely, in real time, economically and efficiently, which assists in accurate resection thus avoiding a thoracotomy, and IU can also detect if there is a potential risk of thrombosis.

## Disclosure

The authors report that there are no conflicts of interest.
